# The Impact of Angiotensin-Converting Enzyme Inhibitors on Immune-Related Adverse Events and Clinical Outcomes in Patients with Metastatic NSCLC

**DOI:** 10.3390/biomedicines14091882

**Published:** 2026-08-24

**Authors:** Noa Shani Shrem, Samer Abu-Rafe, Abed Agbarya, Ashraf Abu Jama, Asmah Miari, Ronen Brenner, Yulia Dudnik, Adan Khalaily, Alexander Yakobson, Samer Hussany, Raya Bdair, Keren Rouvinov, Nashat Abu Yasin, Natalie Maimon Rabinovich, Walid Shalata

**Affiliations:** 1 The Legacy Heritage Cancer Center, Larry Norton Institute, Soroka Medical Center, Beer Sheva 84105, Israel; noashs@clalit.org.il (N.S.S.);; 2 Faculty of Health Sciences, Ben Gurion University of the Negev, Beer Sheva 84105, Israel; 3 Department of Internal Medicine, Soroka Medical Center, Beer Sheva 84105, Israel; 4 Department of Oncology, Bnai Zion Medical Center, Haifa 31048, Israel; 5 Faculty of Medicine, Tel Aviv University, Tel Aviv 69978, Israel; 6 Department of Oncology, Meir Medical Center, Kfar Saba 44180, Israel; 7 Edith Wolfson Medical Center, Oncology Institute, Holon 58220, Israel

**Keywords:** angiotensin-converting enzyme inhibitors, immune-related adverse events, lung cancer, immunotherapy

## Abstract

**Background:** The integration of immune checkpoint inhibitors (ICIs) has transformed metastatic non-small cell lung cancer (NSCLC) therapy. However, the immunomodulatory influence of concurrent medications, including angiotensin-converting enzyme inhibitors (ACEIs), remains poorly defined in real-world practice. **Methods:** This retrospective observational study included 452 patients with metastatic NSCLC treated with first-line ICI-based therapy (ICI monotherapy or chemo-immunotherapy) between 2017 and 2025. Patients were stratified according to chronic ACEI exposure, defined as administration for >2 years. Primary endpoints were overall survival (OS), progression-free survival (PFS), and immune-related adverse events (irAEs). **Results:** Among 452 patients, 71 (15.7%) were chronic ACEI users. ACEI use was associated with significantly longer median OS in univariable analysis (17.0 vs. 14.0 months; HR = 0.78, 95% CI: 0.61–0.99; *p* = 0.047) and a favorable trend toward improved PFS (14.0 vs. 11.0 months; HR = 0.81, 95% CI: 0.64–1.02; *p* = 0.066). ACEI users had higher rates of cutaneous rash (25.4% vs. 13.1%; *p* = 0.011) and transaminase elevation (29.6% vs. 8.9%; *p* < 0.001). Severe grade 3–5 irAEs remained uncommon and did not differ significantly between groups (*p* > 0.05). **Conclusions:** Chronic ACEI use was associated with longer OS in univariable analysis, but this association was not statistically significant after multivariable adjustment. ACEI use was also associated with increased low-grade cutaneous and hepatic toxicities without a significant increase in severe irAEs. Prospective studies are warranted to clarify the clinical significance of these associations.

## 1. Introduction

Lung cancer continues to represent the most significant challenge in global oncology, characterized by its high mortality rate and substantial histological diversity. The disease is primarily categorized into Non-Small Cell Lung Cancer (NSCLC), accounting for approximately 85% of cases, and Small Cell Lung Cancer (SCLC), which is distinguished by its rapid doubling time and early metastatic spread [1,2]. For patients presenting with metastatic disease—stage IV—the therapeutic landscape has shifted from traditional platinum-based chemotherapy toward precision medicine and immune checkpoint inhibitors (ICIs). These therapies, which target the PD-1/PD-L1 and CTLA-4 axes, have demonstrated the ability to induce durable responses and extend overall survival in the metastatic setting [3].

Despite the clinical efficacy of ICIs, their use is frequently complicated by immune-related adverse events (irAEs). These toxicities result from a loss of self-tolerance, leading to inflammatory damage across diverse organ systems, most notably the lungs (pneumonitis), colon (colitis), and endocrine glands [4]. The management of these events is critical, as severe toxicities can necessitate the permanent discontinuation of life-prolonging therapy. Emerging clinical evidence suggests a paradoxical “toxicity–efficacy” link, where the occurrence of certain irAEs may serve as a surrogate marker for enhanced anti-tumor immune activity and improved patient outcomes [5].

Parallel to these oncological developments, increasing attention has been directed toward the potential interaction between concomitant medications and immunotherapy, including angiotensin-converting enzyme inhibitors (ACEIs). ACEIs are widely prescribed for hypertension and cardiovascular comorbidities in patients with lung cancer. Unlike renin–angiotensin system inhibitors that act downstream of angiotensin-converting enzyme (ACE), ACEIs directly inhibit ACE, thereby reducing the conversion of angiotensin I to angiotensin II while simultaneously increasing the availability of bradykinin. This dual effect may be particularly relevant to immune regulation, as both the angiotensin II and bradykinin pathways participate in inflammatory and vascular signaling. Preclinical and epidemiological studies have suggested that ACE inhibition may influence inflammatory cytokines and immune-mediated processes, including pathways involving IL-6 and TNF-α, which have also been implicated in the development of irAEs [6,7,8]. In addition, previous studies have reported associations between long-term ACEI exposure and lung cancer incidence, although the underlying mechanisms remain uncertain [6,7]. These observations have raised the possibility that ACEIs may influence the inflammatory milieu in patients receiving ICIs, potentially affecting both treatment efficacy and toxicity [9,10]. However, clinical evidence regarding this association remains limited and conflicting, with some studies suggesting a potential effect of ACEIs on immune-mediated inflammation and others reporting differences in outcomes among patients receiving PD-1/PD-L1 inhibitors. Importantly, whether these observations represent a direct immunomodulatory effect of ACE inhibition or reflect underlying patient characteristics and comorbidities remains unclear. Therefore, the clinical impact of chronic ACEI exposure in patients treated with ICI-based therapy warrants further investigation.

## 2. Materials and Methods

### 2.1. Patient Enrolment and Data Collection

This retrospective, observational, non-interventional cohort study investigated patients with histologically confirmed, wild-type metastatic NSCLC (absence of an identified actionable driver alteration). The study population consisted of individuals treated in the first-line setting with either ICI monotherapy (e.g., pembrolizumab) or dual ICI combinations (e.g., nivolumab plus ipilimumab), with or without concurrent platinum-based chemotherapy. The study period spanned from January 2017 to September 2025, with a final follow-up cutoff in November 2025.

Electronic medical records were reviewed to extract comprehensive data, including patient demographics (age, sex, smoking history), clinicopathological characteristics (histological subtype, PD-L1 expression, ECOG performance status), and treatment specifics (regimen type, duration, and dates of initiation/discontinuation).

### 2.2. Inclusion and Exclusion Criteria

Adult patients (age ≥ 18 years) were eligible if they received ICIs for metastatic disease and possessed complete records regarding baseline characteristics and medication history. ACEI exposure was strictly defined as documented, continuous administration for at least two years prior to the initiation of immunotherapy, in order to identify patients with established and sustained ACEI exposure before cancer treatment.

To minimize the confounding impact of “immortal time bias” and early mortality from hyper-progressive disease, a landmark inclusion criterion was set: patients must have achieved a Progression-Free Survival (PFS) of at least four months. This allowed for a more reliable evaluation of chronic ACEI influence on both late-onset toxicities and sustained therapeutic response. Patients were excluded if they had incomplete documentation regarding ACEI dosing, unclear immunotherapy records, or were lost to follow-up before the primary endpoints could be assessed.

### 2.3. Definitions of Endpoints

The primary clinical endpoints were Overall Survival (OS), PFS, and the incidence and severity of irAEs.

IrAEs were identified through clinical documentation and graded according to the Common Terminology Criteria for Adverse Events (CTCAE) version 5.0.

Specific focus was placed on the correlation between ACEI use and the development of different toxicities.

### 2.4. Statistical Analysis

Baseline characteristics were compared between the ACEI-exposed group and the non-exposed group. Categorical variables were analyzed using the Chi-square or Fisher’s exact test, while continuous variables were presented as medians (range) and compared using the Mann–Whitney U test. Survival curves for OS and PFS were generated using the Kaplan–Meier method, with differences evaluated via the log-rank test. To isolate the independent effect of ACEIs on immunotherapy outcomes and toxicity risk, multivariable Cox proportional hazards regression models were employed. These models adjusted for potential confounders, including age, sex, smoking status, histology, PD-L1 expression, and ICI regimen. Results are reported as Hazard Ratios (HRs) with 95% Confidence Intervals (CIs). A two-sided *p*-value < 0.05 was considered statistically significant. All statistical analyses were performed using SPSS version 28. Categorical variables were compared using the chi-square test or Fisher’s exact test, as appropriate.

## 3. Results

A total of 452 patients were included, of whom 71 (15.7%) received ACEIs and 381 (84.3%) did not. The median age of the overall cohort was 68 years (range, 37–87). Patients in the ACEI group were older, with a median age of 70 years (range, 53–83), compared with 67 years (range, 37–87) in the non-ACEI group.

The cohort was predominantly male (70.4%), with females comprising 29.6% of patients. The sex distribution was similar between groups, with 32.4% females in the ACEI group and 29.1% in the non-ACEI group.

Baseline performance status was comparable across groups. ECOG performance status was 0 in 27.7% of patients, 1 in 53.3%, and 2–3 in 18.8%. A slightly higher proportion of ACEI users had ECOG 0 (33.8% vs. 26.5%), while ECOG 2–3 was less frequent in this group (15.5% vs. 19.4%).

Regarding smoking status, 48.7% of patients were current smokers, 37.2% were former smokers, and 13.7% were never smokers. The ACEI group included a higher proportion of current smokers (54.9% vs. 47.5%) and fewer former smokers (29.6% vs. 38.6%), while the proportion of never smokers was similar between groups.

Adenocarcinoma was the predominant histologic subtype, observed in 68.1% of patients, whereas 31.9% had squamous cell carcinoma. Histologic distribution was generally balanced between groups, although adenocarcinoma was slightly more frequent among ACEI users (71.8% vs. 67.5%).

PD-L1 expression < 1% was reported in 31.9% of patients, while 68.1% had PD-L1 ≥ 1%. These proportions were similar between groups, with minimal differences between ACEI users and non-users.

With respect to immunotherapy regimen, pembrolizumab was administered in 58.8% of patients overall and was more frequently used in the ACEI group (73.2% vs. 56.2%). In contrast, the combination of nivolumab plus ipilimumab was less commonly used among ACEI users (26.8% vs. 42.3%).

Most patients received chemotherapy in combination with immunotherapy (81.9%), whereas 18.1% were treated with immunotherapy alone. Treatment patterns were broadly similar between groups, although immunotherapy monotherapy was slightly more common among ACEI users (21.1% vs. 17.6%), (Table 1).

**Figure 1:**
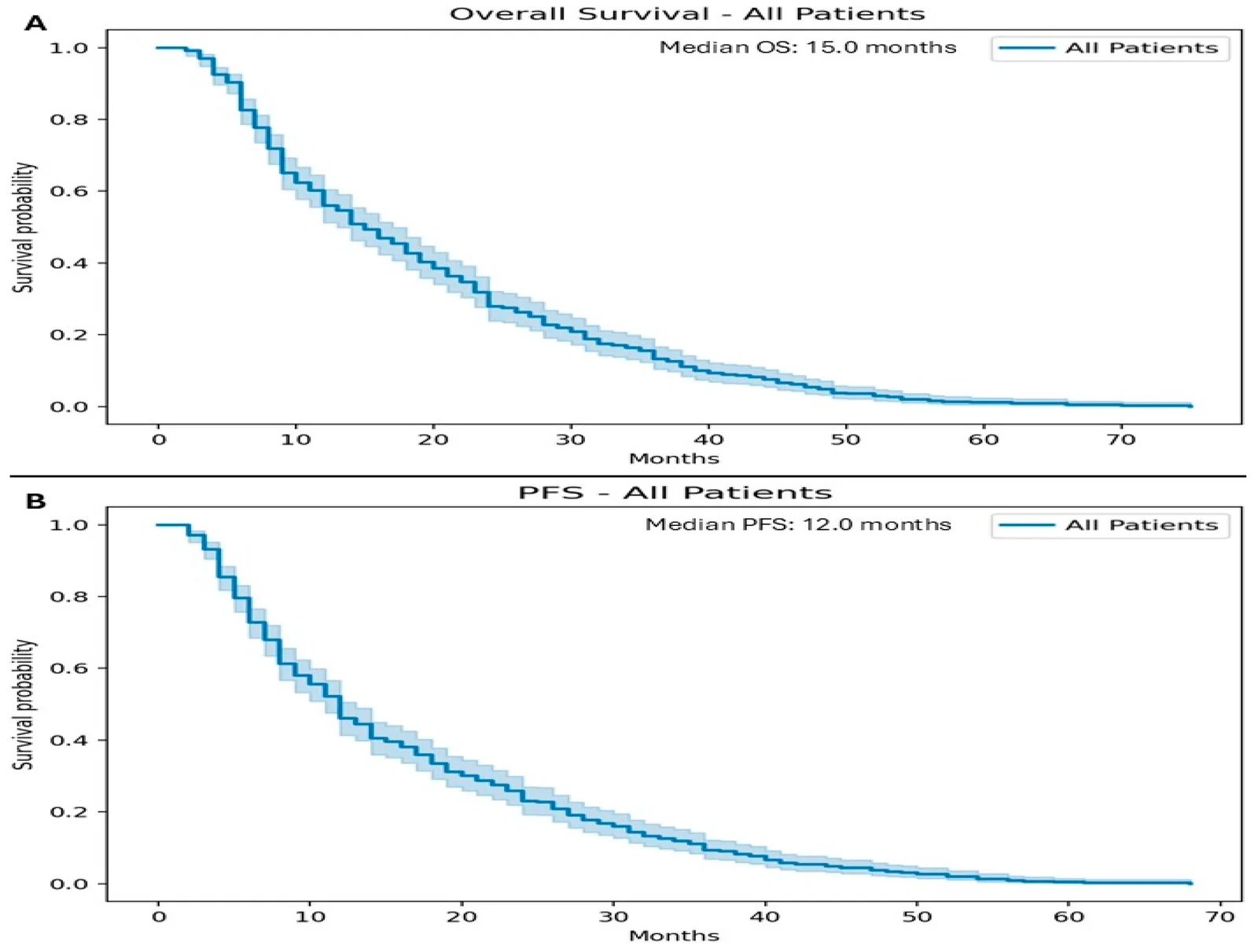
Kaplan–Meier curves illustrating overall survival (**A**) and progression-free survival (**B**) for the entire cohort.

In the overall cohort, the median OS was 15.0 months (Figure 1A), while the median PFS was 12.0 months (Figure 1B).

**Figure 2:**
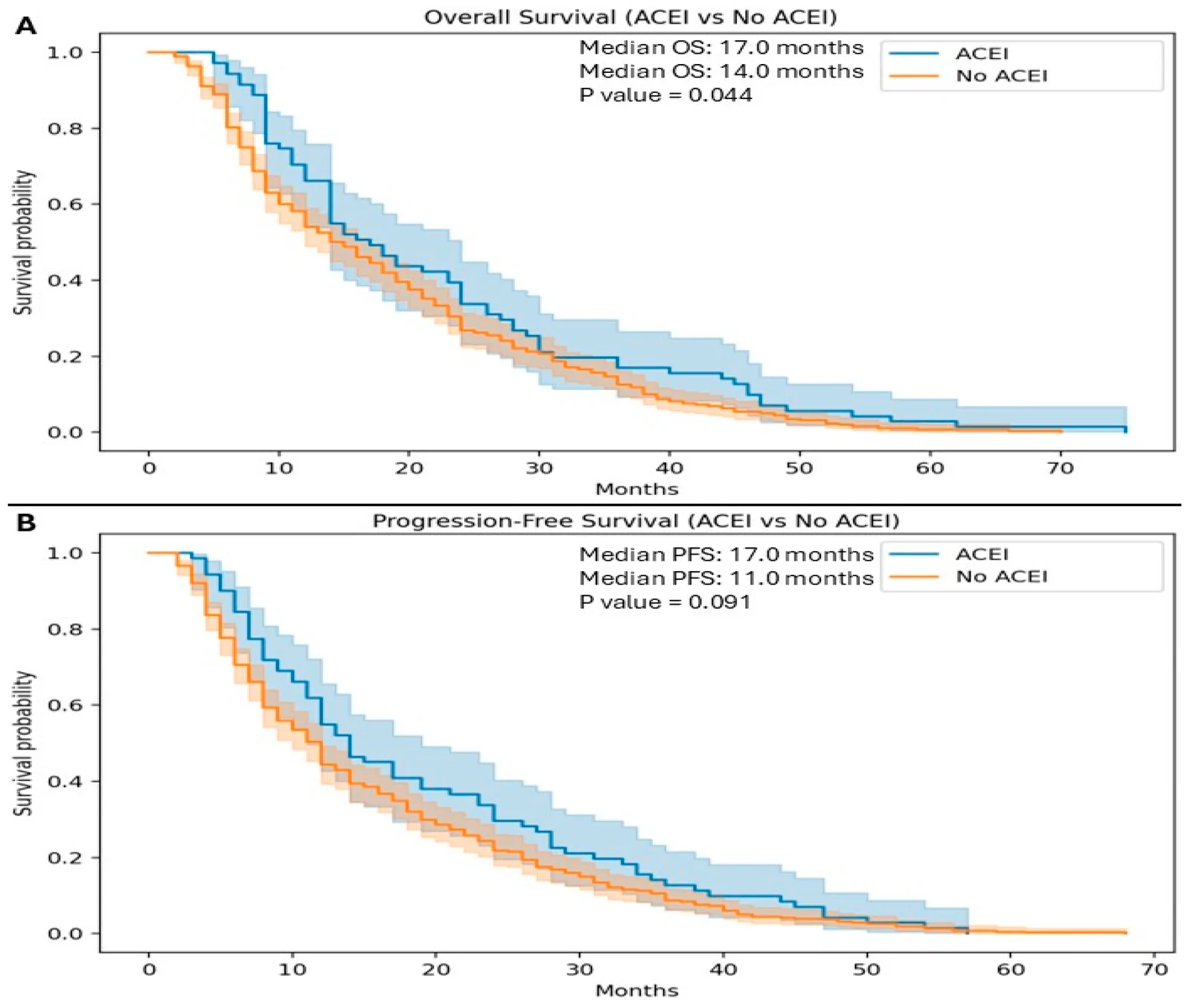
(**A**) Overall Survival: The median overall survival was 17.0 months for patients who received ACEI as chronic medication, and 14.0 months for those with did not received ACEI as chronic medications. The log-rank test showed a statistically significant difference in overall survival between the groups (*p* < 0.047). (**B**) Progression-Free Survival: The median progression-free survival was 14.0 months for patients who received ACEI as chronic medication, and 11.0 months for those who did not received ACEI as chronic medication. The log-rank test demonstrated a trend for statistically significant difference in progression-free survival between the groups (*p* = 0.066).

When stratified by ACEI use, patients receiving ACE inhibitors demonstrated improved outcomes compared with those not receiving ACEIs. The OS was 17.0 months in the ACEI group versus 14.0 months in the non-ACEI group, with this difference reaching statistical significance (log-rank *p* = 0.047). Similarly, median PFS was longer among ACEI users (14.0 months) compared with non-users (11.0 months), although this difference did not reach statistical significance and showed only a trend (log-rank *p* = 0.066), (Figure 2).

The incidence of irAEs was comparable between the two groups for most toxicities, although some clinically relevant differences were observed.

Fatigue was the most frequently reported adverse event, occurring in 318 patients overall (70.4%), with similar rates between the ACEI and non-ACEI groups (73.2% vs. 69.8%, *p* = 0.671). Cutaneous toxicity was significantly more common in patients receiving ACEIs, with rash observed in 25.4% compared with 13.1% in the non-ACEI group (*p* = 0.011), while pruritus showed no significant difference between groups (12.7% vs. 13.1%, *p* = 1.000).

Hepatic toxicity demonstrated the most pronounced association with ACEI exposure. Transaminase elevation occurred in 55 patients overall (12.2%) but was significantly higher in the ACEI group compared with the non-ACEI group (29.6% vs. 8.9%, *p* < 0.001). Bilirubin elevation remained rare and comparable between groups (1.4% vs. 0.8%, *p* = 0.496).

Pulmonary toxicity, including pneumonitis, was observed in 56 patients (12.4%), with a numerically higher but non-significant incidence in the ACEI group (16.9% vs. 11.5%, *p* = 0.238). Endocrine toxicities were frequent but balanced between groups, including hypothyroidism (25.4% vs. 19.4%, *p* = 0.263) and hyperthyroidism (0.0% vs. 3.7%, *p* = 0.141).

Gastrointestinal and renal toxicities, such as diarrhea (15.9% overall), nausea (1.5%), and creatinine elevation (8.2%), did not differ significantly between groups. Rare immune-related events, including myocarditis, esophagitis, pancreatitis, nephritis, and fatal outcomes, were observed at very low frequencies and showed no statistically significant differences between cohorts, (Table 2).

For grade 3–4, irAEs were uncommon overall and demonstrated no statistically significant differences between patients receiving ACEI and those not receiving ACEI therapy. The most frequent severe irAE was fatigue, occurring in 28 patients (6.2%), including 3 patients (4.2%) in the ACEI cohort and 25 patients (6.6%) in the non-ACEI cohort (*p* = 0.597). Elevated transaminases were reported in 14 patients (3.1%) and occurred numerically more often among ACEI users (5.6% vs. 2.6%); however, this difference did not reach statistical significance (*p* = 0.251). Similarly, grade 3–4 diarrhea occurred in 13 patients (2.9%) with no difference between groups (2.8% vs. 2.9%, *p* = 1.000).

Among endocrine toxicities, hypothyroidism was observed in 10 patients (2.2%) and was numerically higher in the ACEI group compared with non-users (4.2% vs. 1.8%), although this was not statistically significant (*p* = 0.197). Severe pneumonitis and pruritus each occurred in 8 patients (1.8%), with comparable rates between the ACEI and non-ACEI groups (both *p* = 0.366). Rash was documented in 7 patients (1.5%) and showed the largest numerical imbalance between groups (4.2% vs. 1.0%), but without statistical significance (*p* = 0.081). Creatinine elevation occurred in 5 patients (1.1%) and was also numerically more frequent in ACEI-treated patients (2.8% vs. 0.8%, *p* = 0.177). Other grade 3–5 irAEs, including arthralgia, bilirubin elevation, syncope, myositis, hyperthyroidism, pancreatitis, nephritis, gastritis, and cardiac events, were rare and each occurred in less than 1.5% of patients, with no statistically significant differences observed between groups. One grade 5 treatment-related death occurred in the non-ACEI cohort. Overall, the incidence of severe irAEs remained low, and ACE inhibitor use was not associated with a statistically significant increase in toxicity (Table 3). For Table 3. *p*-values were not calculated because no formal comparisons were performed.

## 4. Discussion

Lung cancer represents a formidable challenge in contemporary oncology, characterized by its high mortality rate and the complexity of its systemic management. While the administration of ICIs has fundamentally transformed the standard of care—offering hope for long-term survival in metastatic cases—the clinical reality is that many of these patients suffer from multiple comorbidities requiring chronic pharmacotherapy [1,2,3]. The potential for drug–drug interactions between these common medications and immunotherapy is a critical yet insufficiently explored frontier in precision medicine. Currently, the impact of concurrent medications on the delicate balance between anti-tumor efficacy and irAEs remains largely unknown. Given that ACEIs are among the most frequently prescribed drugs for cardiovascular management in this demographic, it is imperative to investigate their immunomodulatory role. Therefore, this study aims to elucidate the clinical interaction between chronic ACEI use and immunotherapy, evaluating how this synergy affects both patient survival and the toxicity profile in a real-world cohort.

The findings of this retrospective cohort study suggest a significant clinical interaction between ACEIs and the efficacy and toxicity profiles of ICIs in metastatic lung cancer. Our analysis of 452 patients revealed that concurrent ACEI use was associated with a statistically significant improvement in OS (17.0 vs. 14.0 months; *p* = 0.047) and a positive trend toward improved PFS (14.0 vs. 11.0 months; *p* = 0.066). These results contribute to an evolving dialogue regarding the use of chronic medications of cardiovascular medications to modulate the tumor microenvironment (TME) and enhance the host’s anti-tumor immune response [11,12,13].

The observed survival advantage in our ACEI-exposed cohort (17.0 months) aligns with a subset of clinical data suggesting that Renin-Angiotensin System (RAS) inhibition may synergize with ICIs. A meta-analysis similarly indicated that patients receiving RAS inhibitors (ACEIs or ARBs) exhibited superior OS and PFS across various malignancies, including NSCLC [12]. However, the existing literature is characterized by significant heterogeneity. For instance, while our study showed a survival benefit, a large retrospective study reported that ACEI use might actually decrease the response rate to PD-1 blockers, particularly in certain histological subtypes [11]. These conflicting results may stem from the “ACEI-paradox”; while ACEIs may theoretically increase lung cancer risk through bradykinin accumulation as noted in early epidemiological studies [6,7], they simultaneously appear to inhibit tumor-promoting inflammation and fibrosis once the disease is established, potentially facilitating better T-cell infiltration.

A primary finding of this research is the significant correlation between ACEI use and specific irAEs, most notably cutaneous rash (25.4% vs. 13.1%; *p* = 0.011) and transaminase elevation (29.6% vs. 8.9%; *p* < 0.001). The substantial increase in hepatic toxicity in the ACEI group is particularly noteworthy, as it suggests that RAS modulation may lower the systemic threshold for immune-mediated organ damage. This observation supports the “toxicity–efficacy” hypothesis, which posits that the development of irAEs is a clinical surrogate for robust immune activation [13,14,15,16]. Patients in our study who experienced these higher rates of toxicity also experienced longer OS, suggesting that the same biological mechanism driving the inflammatory “off-target” effects (rash and hepatitis) may also be responsible for the “on-target” anti-tumor effect [17,18].

In contrast to the all-grade findings, severe (Grade 3–4) irAEs were uncommon overall and demonstrated no statistically significant differences between patients receiving ACEI and those not receiving ACEI therapy. Our data reveal that the most frequent severe irAE was fatigue, occurring in 6.2% of the overall cohort, with statistically comparable distributions between groups (4.2% ACEI vs. 6.6% non-ACEI; *p* = 0.597). While notable numerical imbalances were observed in Grade 3–4 transaminase elevation (5.6% vs. 2.6%; *p* = 0.251) and Grade 3–4 cutaneous rash (4.2% vs. 1.0%; *p* = 0.081), neither crossed the threshold for traditional statistical significance. This lack of statistical differentiation in high-grade toxicity stands in alignment with recent literature reviews, which suggest that while concurrent cardiovascular drugs can shift low-grade inflammatory expression, they rarely exacerbate toxicities to a life-threatening or severe degree [5,10,18,19]. Crucially, the only documented Grade 5 treatment-related death occurred in the non-ACEI cohort, confirming that ACE inhibitor use does not introduce unsafe levels of severe immune-mediated toxicity.

The potential mechanism (our hypothetical) for the survival benefit involves the inhibition of Angiotensin II (AngII). AngII is known to promote an immunosuppressive TME by inducing the recruitment of Myeloid-Derived Suppressor Cells (MDSCs) and stimulating the release of Transforming Growth Factor-beta (TGF-\beta), which hinders T-cell function [5,14,18,19]. By blocking ACE, these immunosuppressive signals are diminished, potentially leading to a more “inflamed” tumor phenotype that is more susceptible to ICIs [19]. We hypothesize that this TME remodeling accounts for the broad-spectrum baseline tissue “priming” observed in our all-grade results. However, RAS signaling is closely interconnected with, but does not directly encompass, the acute inflammatory pathways involved in severe immune-mediated tissue injury. Consequently, RAS inhibition may influence susceptibility to immune-related toxicity without necessarily being sufficient to drive the extensive inflammatory response required for Grade 3–4 organ toxicity. Intracellular cross-regulatory mechanisms may partly explain why the increased hazard observed for mild toxicity did not translate into a statistically significant increase in high-grade irAEs, although this proposed mechanism remains speculative [5,18,19,20,21,22].

Moreover, our findings should be interpreted within the broader context of emerging research evaluating ICI outcomes in clinically complex and historically underrepresented patient populations. Recent systematic reviews have demonstrated that ICIs remain both effective and generally safe in people living with HIV and NSCLC, with survival outcomes and rates of immune-related adverse events comparable to those reported in the general population. These observations support the growing recognition that patient-related factors—including comorbidities, immune status, and concomitant medications—may influence treatment outcomes but do not necessarily preclude successful immunotherapy. In this regard, our study extends the current literature by examining chronic ACEI exposure as a potential modifier of ICI efficacy and toxicity in a large real-world cohort. While we observed a favorable univariable association with survival and a distinct toxicity profile among ACEI users, the attenuation of this association in multivariable analyses underscores the complexity of disentangling medication effects from underlying patient characteristics and comorbidity burden [23].

This study has several limitations that should be considered when interpreting our findings. First, its retrospective and observational design inherently limits the ability to establish causality and may be subject to selection bias, information bias, and residual confounding. Although we adjusted for several clinically relevant baseline characteristics in the multivariable analyses, unmeasured or incompletely measured factors may have influenced both ACEI exposure and clinical outcomes. Second, ACEI exposure was defined according to chronic use for more than 2 years; however, information regarding treatment adherence, dose, specific ACEI agent, and changes in ACEI therapy during the course of cancer treatment was not consistently available, in addition the main cause for receiving ACEI was not mentioned. Third, the use of a ≥4-month PFS criterion may have introduced selection bias by excluding patients who experienced early disease progression or death. This criterion was selected to allow sufficient time for the potential therapeutic effect of ICI-based treatment to become clinically evident; however, it may preferentially select patients with a more favorable early treatment course. Furthermore, survival time was calculated from treatment initiation rather than from the 4-month point, and therefore this approach should not be considered a conventional landmark analysis. Fourth, the multicenter nature of the study may have resulted in heterogeneity in clinical practice, treatment selection, assessment of response, and documentation of immune-related adverse events. Finally, although our findings suggest a potential association between chronic ACEI exposure and outcomes during ICI-based therapy, no direct assessment of the tumor microenvironment, immune-cell populations, or inflammatory biomarkers was performed. Therefore, the biological mechanisms underlying the observed associations remain speculative. These limitations highlight the need for prospective studies with standardized exposure definitions, comprehensive clinical and immunologic data, and appropriately designed landmark analyses to validate our findings.

## 5. Conclusions

This study demonstrates that metastatic lung cancer patients on chronic ACEI therapy experience significantly improved overall survival when treated with ICIs, alongside a higher incidence of specific all-grade toxicities. Reassuringly, this medication did not elevate the risk of severe Grade 3–5 events. Given the high prevalence of hypertension in this patient population, these findings suggest that the choice of antihypertensive therapy may play a beneficial supportive role in determining the success of immunotherapy without compromising patient safety.

## Figures and Tables

**Table 1:** Baseline characteristics according to ACEI use.

Characteristic	Overall (*n* = 452)	ACEI (*n* = 71)	No ACEI (*n* = 381)	*p*-Value
**Age (years)**, *median* (*range*)	68 (37–87)	70 (53–83)	67 (37–87)	
**Gender**, *n* (%)				0.581
Female	134 (29.6%)	23 (32.4%)	111 (29.1%)	
Male	318 (70.4%)	48 (67.6%)	270 (70.9%)	
**ECOG Performance Status**, *n* (%)				0.417
ECOG 0	125 (27.7%)	24 (33.8%)	101 (26.5%)	
ECOG 1	241 (53.3%)	36 (50.7%)	205 (53.8%)	
ECOG 2–3	85 (18.8%)	11 (15.5%)	74 (19.4%)	
**Smoking Status**, *n* (%)				0.368
Current	220 (48.7%)	39 (54.9%)	181 (47.5%)	
Past	168 (37.2%)	21 (29.6%)	147 (38.6%)	
Never	62 (13.7%)	10 (14.1%)	52 (13.6%)	
**Histology**, *n* (%)				0.467
Adenocarcinoma	308 (68.1%)	51 (71.8%)	257 (67.5%)	
Squamous cell carcinoma	144 (31.9%)	20 (28.2%)	124 (32.5%)	
**PD-L1 Expression**, *n* (%)				0.916
PD-L1 < 1%	144 (31.9%)	23 (32.4%)	121 (31.8%)	
PD-L1 ≥ 1%	308 (68.1%)	48 (67.6%)	260 (68.2%)	
**Treatment Regimen**, *n* (%)				0.011
Pembrolizumab	266 (58.8%)	52 (73.2%)	214 (56.2%)	
Nivolumab + Ipilimumab	180 (39.8%)	19 (26.8%)	161 (42.3%)	
**Chemotherapy**, *n* (%)				0.477
Yes	370 (81.9%)	56 (78.9%)	314 (82.4%)	
No	82 (18.1%)	15 (21.1%)	67 (17.6%)	

Abbreviation: *n*, number of patients; ACEI, angiotensin-converting enzyme inhibitor; ECOG, Eastern Cooperative Oncology Group; PD-L1, programmed death-ligand 1.

**Table 2:** Immune-Related Adverse Events According to ACEI Use.

Adverse Event	Overall (*n* = 452)	ACEI (*n* = 71)	No ACEI (*n* = 381)	*p*-Value
Fatigue	318 (70.4%)	52 (73.2%)	266 (69.8%)	0.671
Rash	68 (15.0%)	18 (25.4%)	50 (13.1%)	0.011
Pruritus	59 (13.1%)	9 (12.7%)	50 (13.1%)	1
Arthralgia	40 (8.8%)	8 (11.3%)	32 (8.4%)	0.493
Infusion reaction	1 (0.2%)	0 (0.0%)	1 (0.3%)	1
Diarrhea	72 (15.9%)	9 (12.7%)	63 (16.5%)	0.483
Pneumonitis	56 (12.4%)	12 (16.9%)	44 (11.5%)	0.238
Transaminases elevation	55 (12.2%)	21 (29.6%)	34 (8.9%)	<0.001
Bilirubin elevation	4 (0.9%)	1 (1.4%)	3 (0.8%)	0.496
Creatinine elevation	37 (8.2%)	4 (5.6%)	33 (8.7%)	0.486
Hyperthyroidism	14 (3.1%)	0 (0.0%)	14 (3.7%)	0.141
Hypothyroidism	92 (20.4%)	18 (25.4%)	74 (19.4%)	0.263
Cough	7 (1.5%)	2 (2.8%)	5 (1.3%)	0.303
Nausea	7 (1.5%)	1 (1.4%)	6 (1.6%)	1
Neutropenia	4 (0.9%)	1 (1.4%)	3 (0.8%)	0.496
Cardiac event	1 (0.2%)	1 (1.4%)	0 (0.0%)	0.157
Orthostatic hypotension/Vertigo	1 (0.2%)	1 (1.4%)	0 (0.0%)	0.157
Hyponatremia	1 (0.2%)	1 (1.4%)	0 (0.0%)	0.157
Esophagitis	1 (0.2%)	1 (1.4%)	0 (0.0%)	0.157
Abdominal pain	6 (1.3%)	0 (0.0%)	6 (1.6%)	0.596
Neuropathy	7 (1.5%)	0 (0.0%)	7 (1.8%)	0.603
Syncope	2 (0.4%)	0 (0.0%)	2 (0.5%)	1
Vomiting	2 (0.4%)	0 (0.0%)	2 (0.5%)	1
Myositis	2 (0.4%)	0 (0.0%)	2 (0.5%)	1
Polyneuropathy	1 (0.2%)	0 (0.0%)	1 (0.3%)	1
Periorbital edema	1 (0.2%)	0 (0.0%)	1 (0.3%)	1
Pancreatitis	1 (0.2%)	0 (0.0%)	1 (0.3%)	1
Nephritis	1 (0.2%)	0 (0.0%)	1 (0.3%)	1
Death	1 (0.2%)	0 (0.0%)	1 (0.3%)	1
Gastritis	1 (0.2%)	0 (0.0%)	1 (0.3%)	1

Abbreviation: *n*, number of patients; ACEI, angiotensin-converting enzyme inhibitor.

**Table 3:** Incidence of Grade 3–5 Immune-Related Adverse Events.

Adverse Event	Overall (*n* = 452)	ACEI (*n* = 71)	No ACEI (*n* = 381)
Fatigue	28 (6.2%)	3 (4.2%)	25 (6.6%)
Transaminases elevation	14 (3.1%)	4 (5.6%)	10 (2.6%)
Diarrhea	13 (2.9%)	2 (2.8%)	11 (2.9%)
Hypothyroidism	10 (2.2%)	3 (4.2%)	7 (1.8%)
Pneumonitis	8 (1.8%)	2 (2.8%)	6 (1.6%)
Pruritus	8 (1.8%)	2 (2.8%)	6 (1.6%)
Rash	7 (1.5%)	3 (4.2%)	4 (1.0%)
Creatinine elevation	5 (1.1%)	2 (2.8%)	3 (0.8%)
Arthralgia	5 (1.1%)	1 (1.4%)	4 (1.0%)
Bilirubin elevation	4 (0.9%)	1 (1.4%)	3 (0.8%)
Syncope	2 (0.4%)	0 (0.0%)	2 (0.5%)
Myositis	2 (0.4%)	0 (0.0%)	2 (0.5%)
Hyperthyroidism	2 (0.4%)	0 (0.0%)	2 (0.5%)
Cardiac event	1 (0.2%)	1 (1.4%)	0 (0.0%)
Pancreatitis	1 (0.2%)	0 (0.0%)	1 (0.3%)
Nephritis	1 (0.2%)	0 (0.0%)	1 (0.3%)
Gastritis	1 (0.2%)	0 (0.0%)	1 (0.3%)
Death (grade 5)	1 (0.2%)	0 (0.0%)	1 (0.3%)

Abbreviation: *n*, number of patients; ACEI, angiotensin-converting enzyme inhibitor.

## Data Availability

The original contributions presented in this study are included in the article. Further inquiries can be directed to the corresponding author.

## Abbreviations

The following abbreviations are used in this manuscript:

ACE	Angiotensin-converting enzyme
ACEI	Angiotensin-converting enzyme inhibitor
AngII	Angiotensin II
ARBs	Angiotensin II receptor blockers
CI	Confidence interval
CTCAE	Common Terminology Criteria for Adverse Events
CTLA-4	Cytotoxic T-lymphocyte-associated protein 4
ECOG	Eastern Cooperative Oncology Group
HR	Hazard ratio
ICI	Immune checkpoint inhibitor
ICIs	Immune checkpoint inhibitors
IL-6	Interleukin-6
irAE	Immune-related adverse event
irAEs	Immune-related adverse events
MDSCs	Myeloid-derived suppressor cells
NSCLC	Non-small cell lung cancer
OS	Overall survival
PD-1	Programmed cell death protein 1
PD-L1	Programmed death-ligand 1
PFS	Progression-free survival
RAS	Renin–angiotensin system
SCLC	Small cell lung cancer
SPSS	Statistical Package for the Social Sciences
TGF-β	Transforming growth factor-beta
TME	Tumor microenvironment
TNF-α	Tumor necrosis factor-alpha
